# Navigating Nutritional Strategies: Permissive Underfeeding in Critically Ill Patients

**DOI:** 10.7759/cureus.58083

**Published:** 2024-04-11

**Authors:** Varun N Thawkar, Karuna Taksande

**Affiliations:** 1 Anaesthesiology, Jawaharlal Nehru Medical College, Datta Meghe Institute of Higher Education and Research, Wardha, IND

**Keywords:** patient outcomes, overfeeding, intensive care unit (icu), nutritional support, critical illness, permissive underfeeding

## Abstract

Nutritional support is a critical component of care for critically ill patients, impacting their recovery and overall prognosis. Traditional approaches to feeding in the intensive care unit (ICU) have focused on meeting estimated energy requirements, often resulting in unintended consequences such as overfeeding and associated complications. Permissive underfeeding, a concept gaining attention recently, offers a more controlled approach by intentionally providing fewer calories than traditionally recommended. This comprehensive review explores the rationale, evidence, and practical considerations surrounding permissive underfeeding in critically ill patients. We discuss the physiological basis of permissive underfeeding, its potential benefits in mitigating the risks of overfeeding, and the challenges associated with implementation in clinical practice. Through an analysis of critical studies and clinical trials, we evaluate the comparative effectiveness of permissive underfeeding versus traditional feeding methods and examine its impact on patient outcomes. Recommendations for patient selection, monitoring, and future research directions are provided to guide clinicians in optimizing nutritional support strategies for critically ill individuals. By considering the role of permissive underfeeding alongside traditional feeding approaches, healthcare professionals can tailor nutritional interventions to individual patient needs, ultimately improving outcomes in the ICU.

## Introduction and background

Permissive underfeeding represents a nuanced departure from the conventional approach to nutritional support in critically ill patients [1]. Rather than aiming to meet or exceed estimated caloric requirements, permissive underfeeding intentionally delivers a reduced calorie intake, often below traditional targets. This approach acknowledges the complex metabolic alterations during critical illness, wherein patients may exhibit decreased energy expenditure and altered nutrient utilization. By permitting a controlled level of nutrient restriction, permissive underfeeding aims to align nutritional support more closely with the dynamic physiological needs of critically ill individuals, potentially mitigating the risks associated with overfeeding while still ensuring essential nutrient delivery [2].

The significance of appropriate nutritional support cannot be overstated in the care of critically ill patients. During acute illness or injury, the body undergoes profound metabolic changes characterized by increased energy expenditure, heightened catabolism, and altered nutrient partitioning. Adequate nutrition is vital for sustaining vital organ function, supporting immune responses, promoting wound healing, and preserving lean body mass [3]. Insufficient or inappropriate nutritional support in critically ill patients can exacerbate malnutrition, impair recovery, prolong hospital stays, increase susceptibility to complications, and ultimately compromise patient outcomes. Therefore, optimizing nutritional strategies tailored to the unique needs of critically ill individuals is paramount for enhancing clinical outcomes and improving overall prognosis [4].

This review explores permissive underfeeding as a strategic approach to nutritional management in critically ill patients. By elucidating the underlying physiological mechanisms and rationale behind permissive underfeeding, examining the clinical evidence supporting its efficacy and safety, and discussing practical considerations for its implementation in the clinical setting, this review aims to provide healthcare professionals with a nuanced understanding of this alternative nutritional strategy. Through a thorough synthesis of existing literature and critical analysis, this review seeks to elucidate the potential benefits and limitations of permissive underfeeding compared to traditional feeding approaches, informing evidence-based decision-making in providing nutritional support for critically ill patients.

## Review

The physiology of critical illness

Metabolic Alterations in Critical Illness

Metabolic changes in critical illness entail a transition towards catabolism, prompting the depletion of glycogen, protein, and fat reserves in critically ill patients. This metabolic shift is influenced by hormonal fluctuations triggered by the activation of the hypothalamic-pituitary axis, leading to the secretion of hormones such as cortisol, thyroid-stimulating hormone, and growth hormone. Moreover, there is a decrease in the levels of the hunger hormone ghrelin, accompanied by elevated levels of peptide YY and cholecystokinin, which foster anorexia in critically ill individuals [5]. Inflammation is pivotal in driving metabolic alterations during critical illness, with cytokines like interleukin-1 and interleukin-6 contributing to the breakdown of proteins and fats. Excessive inflammation can induce oxidative stress, causing harm to cellular components such as lipid membranes, DNA, and proteins. Damage to mitochondria due to oxidative stress can impair energy production and cellular functions, thereby affecting patient prognosis [5]. Furthermore, critical illness precipitates metabolic imbalances typified by a shift towards catabolism, leading to the depletion of glycogen, protein, and fat stores. Patients may endure a net negative nitrogen balance, experiencing substantial loss of lean body mass daily. Insulin resistance emerges as vital organs prioritize nutrient absorption over peripheral tissues, contributing to the hyperglycemia commonly observed in acute illness [5].

Impact of Stress Response on Nutritional Requirements

The stress response profoundly impacts nutritional requirements by modulating the body's metabolic pathways and intensifying the demand for specific nutrients. When confronted with stress, a cascade of reactions occurs, releasing hormones like adrenaline, noradrenaline, and cortisol. While these hormones are vital for human function, their chronic elevation can lead to physical and mental disturbances. Excessive or prolonged stress can induce detrimental effects on various organs, resulting in tissue damage over time and influencing the regulation of cortisol production [6]. Chronic stress, particularly, is associated with a spectrum of disorders and diseases, encompassing mental health disturbances, cardiovascular diseases, metabolic disorders such as obesity and diabetes, sleep disturbances, and certain cancers. The adverse effects of stress on the body are linked to elevations in inflammatory markers, oxidative stress, disturbances in mitochondrial function, and disruptions in thyroid and sex hormone regulation. Exposure to stress and the consequent overproduction of cortisol can contribute significantly to these deleterious effects on the body [7]. Furthermore, alterations in nutrient status during periods of stress may heighten the risk of diseases, as more significant reserves of nutrients may be necessary during elevated mental or physical demands. Essential nutrients such as vitamins, minerals, proteins, and fatty acids play critical roles in enzymatic processes, energy generation, immune function, hormone synthesis, tissue regeneration, and other vital biological functions. Recognizing the impact of stress on micronutrient levels is crucial for understanding how stress influences overall health and susceptibility to disease, underscoring the importance of maintaining adequate nutrient levels during stressful episodes [8].

Role of Inflammation and Catabolism

In chronic critical illness (CCI), understanding the interplay between inflammation and catabolism is pivotal in elucidating the pathophysiology of this condition. Persistent inflammation, immunosuppression, and catabolism syndrome (PICS) has emerged as a mechanistic framework to elucidate the genesis of CCI in critically ill individuals. This syndrome delineates a cyclical process of chronic low-grade inflammation, immunosuppression, and catabolism, culminating in substantial metabolic deviations and compromised immune functionality [9-11]. The enduring inflammation witnessed in patients afflicted with CCI is marked by heightened serum levels of inflammatory markers such as interleukin-6 and the continual release of endogenous alarmins, fostering chronic inflammation and organ damage. This protracted inflammatory milieu can precipitate alterations in bone marrow function, chronic anemia, lymphopenia, and muscle wasting [12]. Furthermore, the immunosuppressive facet of PICS encompasses lymphocyte dysfunction, diminished antigen presentation, and expansion of myeloid-derived suppressor cells (MDSCs), all of which contribute to perpetuating inflammation and muscle wasting [10,11]. Conversely, catabolism in CCI patients entails impairments in carbohydrate, lipid, and protein metabolism. This sustained catabolic state fosters muscle wasting, cachexia, and loss of lean body mass akin to conditions observed in cancer and other chronic inflammatory disorders. The confluence of persistent inflammation, immunosuppression, and catabolism presents a formidable clinical challenge characterized by prolonged stays in the intensive care unit (ICU), heightened resource utilization, suboptimal long-term outcomes, and escalated mortality rates among patients grappling with CCI [9,11].

Traditional nutritional strategies in critically ill patients

Standard Caloric Provision

Standard caloric provision for critically ill patients is subject to variation, contingent upon factors such as nutritional vulnerability, body mass index (BMI), and individual requisites. In the absence of indirect calorimetry (IC), guidelines advocate the utilization of weight-based equations to approximate caloric necessities. For patients possessing a BMI falling within the range of 30-50, it is recommended to administer 11-14 kcal/kg of actual body weight (ABW) per day. Conversely, for individuals with a BMI surpassing 50, the recommended intake ranges from 22-25 kcal/kg of ideal body weight (IBW) per day. Regarding protein consumption, a minimum of 2.0 gm/kg of IBW per day is advised for patients with a BMI ranging from 30-40, escalating to 2.5 gm/kg of IBW per day for those with a BMI exceeding 40 [13]. Furthermore, enteral nutrition (EN) is favored over parenteral nutrition (PN) in critically ill patients, necessitating nutritional support therapy. Prompt initiation of EN within 24-48 hours is advocated for patients incapable of sustaining volitional intake, particularly those deemed to be in elevated nutritional jeopardy or significantly malnourished. Protocols delineating enteral feeding should be tailored to elevate the overall percentage of goal calories dispensed to furnish over 80% of estimated protein and energy requisites within the initial 48-72 hours. PN induction is recommended upon admission for patients categorized as high nutritional risk or severely malnourished in circumstances where EN is not viable. Additionally, there is a suggestion to complement with PN if enteral routes fail to fulfill more than 60% of energy and protein demands after 7-10 days [14].

Challenges and Limitations

**Figure 1:**
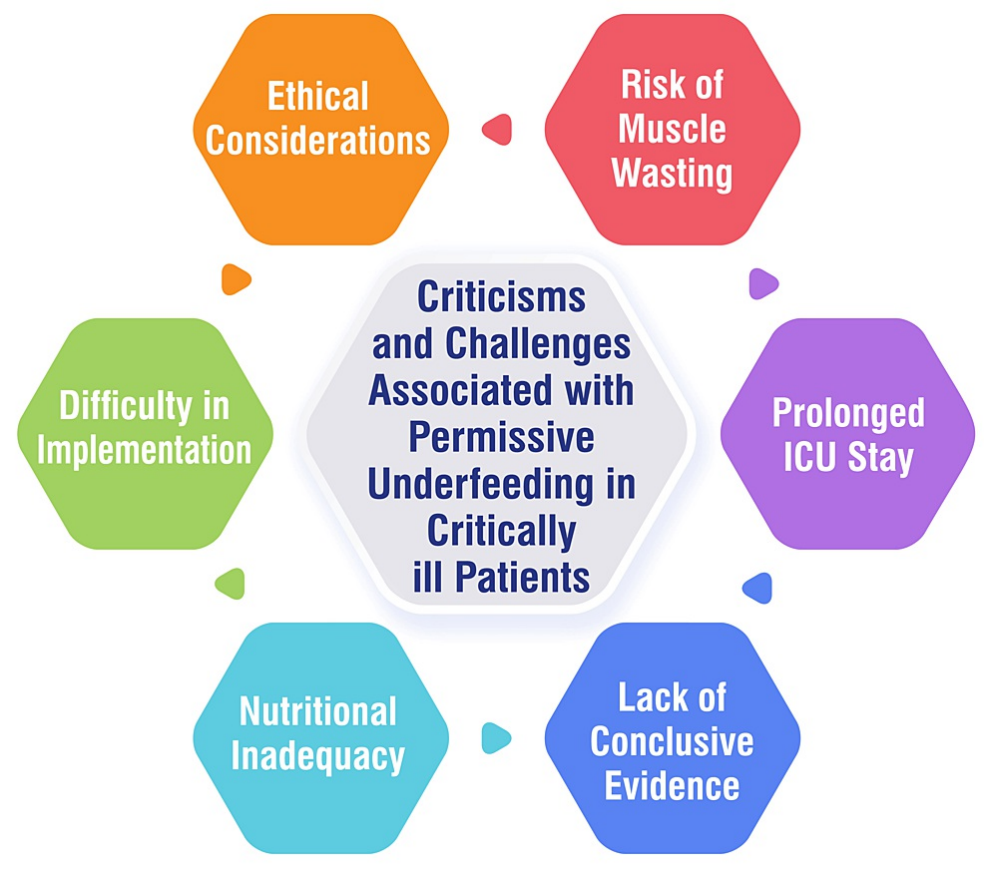
Criticisms and challenges associated with permissive underfeeding in critically ill patients ICU: Intensive care unit Image credit: Varun N. Thawkar

The unpredictable clinical course of patients poses a significant challenge in ensuring adequate nutrition support. Even seemingly stable patients can experience sudden changes, leading to energy and protein deficits that complicate meeting their nutritional requirements [15]. Healthcare providers may exhibit reluctance to perform additional procedures for enteral access, impeding the delivery of necessary nutrition therapy to critically ill patients [15]. High ventilatory demands, particularly prevalent in patients with conditions like COVID-19, can further complicate the provision of nutrition therapy and hinder the ability to fulfill energy requirements [15]. Additionally, intensivists may hesitate to administer supplemental PN, fearing a lack of perceived benefits, thereby complicating the delivery of sufficient energy and nutrients to patients [15]. Gastrointestinal dysfunction, a common occurrence during critical illness, significantly affects the provision and absorption of nutrition therapy. Challenges such as altered digestion and absorption of EN, changes in gut mucosal integrity, and mesenteric perfusion issues can all impede the delivery of optimal nutrition support [16]. Traditional approaches to nutrition therapy, relying on intragastric infusion of EN, have demonstrated limited success in meeting the energy and protein needs of critically ill patients, particularly those with COVID-19. Self-reported data suggests that only a small proportion of patients had their full nutritional requirements met, with many receiving less than 60% of goal feeds [16]. Addressing these challenges necessitates the development of protocols that optimize EN delivery, consider early postpyloric infusion, and address the potential requirement for supplemental PN in deteriorating nutritional statuses. Overcoming these barriers is imperative for improving outcomes and redefining nutrition therapy's role in critically ill patients' care. Criticisms and challenges associated with permissive underfeeding in critically ill patients are shown in Figure 1.

Potential Adverse Effects of Overfeeding

Overindulgence can precipitate numerous adverse effects on the body, particularly concerning metabolic health and overall well-being. Prolonged overconsumption often culminates in obesity, a pivotal risk factor for metabolic syndrome, a constellation of conditions including heart disease, diabetes, and stroke. Metabolic syndrome is characterized by elevated blood pressure, insulin resistance, inflammation, and heightened circulating fat levels. Insulin resistance, intricately linked to chronic overeating, poses a considerable risk of progressing to type 2 diabetes if left unmanaged [17]. Furthermore, habitual overeating may exert long-term repercussions on brain function. Research indicates a correlation between persistent overindulgence, obesity, and cognitive decline, especially among older adults. Evidence suggests that being overweight is associated with detrimental effects on memory when compared to individuals with normal weight. While additional research is warranted to comprehensively grasp the extent and mechanisms of mental decline linked to overeating and obesity, integrating healthy fats such as avocados, nut butter, fatty fish, and olive oil into one's diet may mitigate cognitive decline [17]. Moreover, overindulgence can instigate physical discomfort, such as nausea and indigestion. When the stomach reaches its capacity due to excessive food intake, nausea or indigestion may manifest. In severe instances, this discomfort can precipitate vomiting as a mechanism for the body to alleviate acute stomach pressure [17].

Permissive underfeeding: concept and rationale

Definition and Principles

Permissive underfeeding in critically ill patients hinges on the premise that excessive nutrient intake can yield adverse effects from both metabolic and functional standpoints. It entails the deliberate restriction of nutrient provision, possibly below estimated requirements, to confer potential benefits. The foundational principles of permissive underfeeding draw from the theory of "hormesis," which posits that a low dose of a substance can exert a favorable effect, whereas higher doses may prove detrimental. This approach seeks to mitigate the complications associated with nutrient overfeeding, particularly during the acute phase of critical illness, by administering reduced energy intake to optimize metabolic regulation and enhance patient outcomes [18].

Physiological Basis

The physiological foundation behind permissive underfeeding in critically ill patients lies in the notion that restricting nutrient intake, even below estimated requirements, could yield benefits by circumventing the adverse effects of overfeeding. This approach is predicated on the understanding that heightened nutrient intake may engender negative consequences from both metabolic and functional perspectives. Permissive underfeeding provides under 40-60% of the calories needed for daily energy expenditure, deliberately curbing energy intake while ensuring adequate protein or fat macronutrients. The overarching goal of this strategy is to optimize patient care by minimizing the risks of morbidity and mortality associated with excessive caloric intake, particularly during the acute phase of critical illness. Empirical evidence suggests that permissive underfeeding may be linked to lower mortality and morbidity rates compared to target feeding in critically ill patients [2,19]. The rationale for adopting this approach stems from recognizing that precise caloric targets for critically ill patients still need to be discovered, and even calorimetry fails to provide exact guidance on optimal energy requirements. By purposefully reducing nutrient intake, permissive underfeeding may, in fact, better align with the requisite rate of feeding for critically ill patients, given the uncertainties surrounding eucaloric feeding targets. Furthermore, a theoretical immunological advantage exists to hypocaloric nutrition, as calorie restriction has been associated with enhanced longevity in animal models and may modulate the inflammatory response during critical illness [20].

Evidence Supporting Permissive Underfeeding

The evidence supporting permissive underfeeding in critically ill patients stems from various studies and systematic reviews, indicating potential benefits across multiple parameters. Research suggests that permissive underfeeding may reduce ICU mortality, diminish the occurrence of gastrointestinal adverse events, and shorten the duration of mechanical ventilation. A comprehensive systematic review and meta-analysis, encompassing 23 randomized controlled trials (RCTs) with 11,444 critically ill patients, revealed that permissive underfeeding significantly lowered ICU mortality, decreased the frequency of gastrointestinal adverse events, and shortened mechanical ventilation duration. However, it did not improve overall mortality, underscoring the necessity for further well-designed, large-scale RCTs to validate these findings [1]. Studies have additionally demonstrated that administering reduced energy intake in critically ill and obese patients can enhance metabolic control, alleviate the adverse effects of overfeeding, and bolster patient outcomes [18,21]. Despite the requisite for additional research, permissive underfeeding presents a promising avenue for optimizing nutritional strategies in critically ill patients.

Clinical considerations for implementing permissive underfeeding

Patient Selection Criteria

The patient selection criteria for studies investigating permissive underfeeding in critically ill patients typically encompass individuals aged 18-80 years, admitted to an ICU, with an anticipated stay of at least 72 hours, and initiated on enteral feeding within 48 hours of ICU admission. Exclusion criteria commonly include factors such as a lack of commitment to ongoing life support, brain death, pre-existing conditions with anticipated high mortality rates, post-cardiac arrest status, utilization of total PN, pregnancy, admission following burns or liver transplant, and patients receiving 'high-dose' vasopressors [21]. These criteria are designed to ensure that the study population comprises critically ill adults who are suitable candidates for or can be evaluated for permissive underfeeding strategies.

Monitoring and Assessing Nutritional Status

Monitoring and assessing the nutritional status of critically ill patients is paramount for their care and ultimate outcomes. Various tools and indicators are employed for this purpose, encompassing anthropometric measurements such as body weight, BMI, mid-arm muscle circumference (MAMC), triceps skinfold thickness (TSF), and calf circumference. Additionally, biochemical markers like total protein, albumin, pre-albumin, and immune competence markers such as lymphocyte count are utilized [22]. Among these tools, subjective global assessment (SGA) stands out as a valuable clinical questionnaire that evaluates the nutritional status of critically ill patients based on their history, obviating the necessity for extensive laboratory data. SGA has demonstrated significant diagnostic value in this patient population [22]. Nutritional screening tools are pivotal in ascertaining the nutritional status of critically ill patients. These tools facilitate early detection of malnutrition, a factor that can significantly influence outcomes such as the duration of ICU/hospitalization and mortality rates. Regular monitoring of feeding patterns, including the type of feeding and macronutrient intake, is indispensable to ensure that patients receive adequate calorie and protein intake throughout their ICU stay [23]. Implementing these assessment tools and monitoring strategies empowers healthcare providers to optimize nutritional support for critically ill patients, potentially enhancing their overall prognosis and facilitating recovery.

Practical Implementation in the Clinical Setting

Patient selection is critical in implementing permissive underfeeding strategies in critically ill individuals. It involves identifying patients who may benefit from this approach, considering factors such as nutritional risk, conditions like acute respiratory distress syndrome (ARDS), and individual nutritional requirements [1,21]. Nutritional assessment plays a pivotal role in guiding permissive underfeeding protocols. A comprehensive nutritional evaluation is conducted to ascertain the appropriate caloric intake, ensuring that protein intake remains sufficient while caloric intake is reduced [1,21]. Regular monitoring of patients' responses to permissive underfeeding is essential. This entails assessing for gastrointestinal adverse events, ICU mortality rates, mechanical ventilation duration, and overall mortality outcomes to gauge the efficacy and safety of the approach [1,21]. A tailored approach to nutritional support is imperative. Nutritional regimens are customized based on individual patient characteristics, and feeding strategies are adjusted to optimize outcomes while mitigating risks associated with underfeeding [1,21]. Acknowledging the need for further research is paramount. Additional well-designed RCTs are warranted to validate the efficacy of permissive underfeeding in critically ill patients and provide more definitive conclusions regarding its impact on mortality and other outcomes [1,21].

Benefits and risks of permissive underfeeding

Potential Advantages

**Figure 2:**
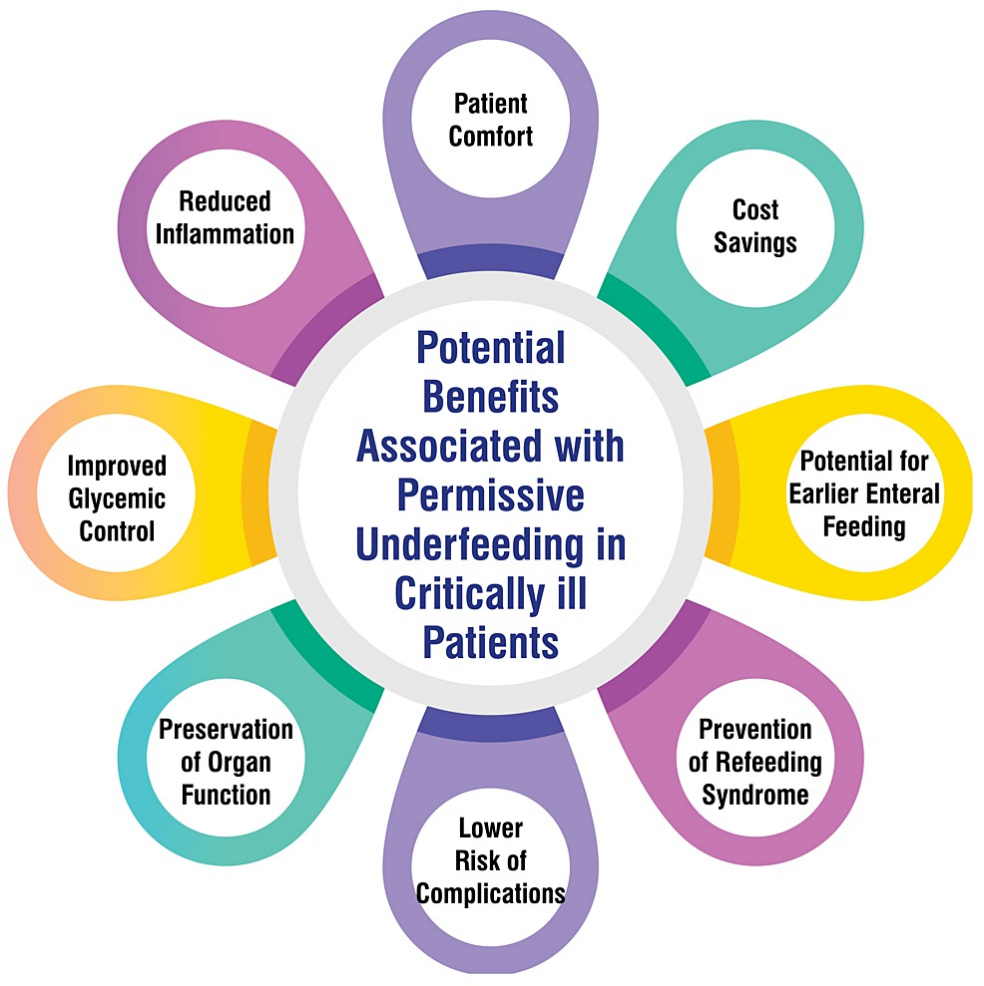
Potential benefits associated with permissive underfeeding in critically ill patients Image credit: Varun N. Thawkar

Permissive underfeeding in critically ill patients presents several potential advantages, as evidenced by research findings. Studies indicate that permissive underfeeding may result in decreased ICU mortality rates, reduced gastrointestinal adverse events, and a shorter duration of mechanical ventilation [18]. Moreover, the concept of permissive underfeeding is rooted in the understanding that higher nutrient intake can prove detrimental both metabolically and functionally, with animal studies demonstrating improved morbidity and mortality outcomes with energy restriction [18]. Additionally, in obese patients, a hypocaloric feeding regimen can foster nitrogen equilibrium and minimize negative nitrogen balance without inducing weight loss, underscoring the potential metabolic benefits of permissive underfeeding in specific patient cohorts [24]. Despite the necessity for further well-designed studies, administering reduced energy intake in critically ill and obese patients may ameliorate metabolic control, alleviate the adverse consequences of overfeeding, and ultimately augment patient outcomes [24]. Potential benefits associated with permissive underfeeding in critically ill patients are shown in Figure 2.

Comparison With Traditional Feeding Approaches

Research has scrutinized the comparison between permissive underfeeding and standard enteral feeding in critically ill patients. One study contrasting permissive underfeeding with standard feeding discovered no notable difference between the cohorts in ICU, hospital, 28-day, or 180-day mortality rates [21]. Conversely, a systematic review and meta-analysis encompassing 23 RCTs unveiled that permissive underfeeding notably diminished ICU mortality, reduced gastrointestinal adverse events, and abbreviated mechanical ventilation duration compared to standard feeding [25,26]. In a separate investigation, patients subjected to permissive underfeeding exhibited outcomes akin to those receiving standard feeding concerning 90-day mortality, duration of mechanical ventilation, length of ICU stay, and incidences of ICU-associated infections [21]. Nevertheless, the permissive underfeeding cohort demonstrated a notable reduction in insulin demand and a lower frequency of gastrointestinal intolerance than the standard feeding group [21]. In sum, while permissive underfeeding may proffer benefits such as decreased ICU mortality and enhanced tolerance, its comparison with conventional feeding methodologies like standard enteral feeding does not invariably reveal superiority across all outcomes. This underscores the imperative for further research to comprehensively grasp the implications of these nutritional strategies in critically ill patients.

Studies and clinical trials evaluating permissive underfeeding

Overview of Key Studies

Critical studies on permissive underfeeding in critically ill patients offer valuable insights into this nutritional strategy. One study elucidates the rationale behind permissive underfeeding, highlighting the potential detriment of higher nutrient intake from both metabolic and functional perspectives. It cites animal studies demonstrating improved morbidity and mortality with energy restriction and discusses how hypocaloric feeding regimens in obese patients can maintain nitrogen equilibrium without inducing weight loss. While limited research exists on permissive underfeeding in critically ill patients, studies on enterally supported patients suggest a correlation between higher caloric intake and decreased morbidity and mortality [18]. Another study, a sub-study of the PermiT trial, analyzed serum samples from critically ill patients to explore the associations of caloric restriction, inflammatory response profiles, and outcomes. The findings revealed that permissive underfeeding was not significantly linked to 90-day mortality or the need for renal replacement therapy. Furthermore, there were no notable differences in serum levels of cytokines between the permissive underfeeding and standard feeding groups [27]. Moreover, a systematic review and trial sequential meta-analysis comprising 23 RCTs with 11,444 critically ill patients demonstrated that permissive underfeeding reduced ICU mortality diminished gastrointestinal adverse events, and shortened mechanical ventilation duration. However, this approach did not significantly enhance overall mortality rates. The study underscores the necessity for well-designed RCTs to validate these conclusions and provide more robust evidence regarding the impact of permissive underfeeding on critically ill patients [1]. These studies underscore the potential benefits of permissive underfeeding in critical care settings, particularly in improving specific outcomes such as ICU mortality and mechanical ventilation duration. However, further research is imperative to corroborate these findings and establish a clearer understanding of the overall impact of permissive underfeeding on critically ill patients.

Comparative Analyses

The PermiT trial, which compared permissive underfeeding with standard enteral feeding, yielded no significant difference in 90-day mortality between the two groups [21]. Similarly, a systematic review and meta-analysis involving 23 RCTs indicated that permissive underfeeding significantly reduced ICU mortality but did not significantly affect overall mortality [1,27]. Notably, the PermiT trial observed a decreased requirement for renal replacement therapy in the permissive underfeeding group, with no serious adverse events reported [21]. The systematic review and meta-analysis also demonstrated a decrease in gastrointestinal adverse events associated with permissive underfeeding [1,27]. Both the PermiT trial and the systematic review highlighted a shorter duration of mechanical ventilation in patients subjected to permissive underfeeding [1,27]. However, the PermiT trial acknowledged limitations such as the inability to blind the intervention and variations in caloric intake between groups [21]. Similarly, the systematic review emphasized the imperative for larger, well-designed RCTs to verify the impact of permissive underfeeding on critically ill patients [1]. These comparative analyses underscore the potential benefits of permissive underfeeding in critical care settings, particularly in reducing ICU mortality and mechanical ventilation duration. Nonetheless, further research is indispensable to validate these findings and furnish more conclusive evidence regarding the overall impact of permissive underfeeding on critically ill patients.

Interpretation of Findings

The evidence gathered from various sources underscores the association between permissive underfeeding in critically ill patients and a range of outcomes. Studies indicate that permissive underfeeding may lead to a reduction in ICU mortality, a decrease in the occurrence of gastrointestinal adverse events, and a shorter duration of mechanical ventilation [1,18,19]. However, it's crucial to recognize that while permissive underfeeding demonstrates benefits in specific outcomes such as ICU mortality and mechanical ventilation duration, it doesn't necessarily significantly improve overall mortality [1,18]. Furthermore, recent RCTs have presented mixed results regarding the advantages of early full feeding in critically ill patients. Some studies have even suggested harm associated with early supplementation of inadequate nutrition, leading to prolonged dependency on ICU care and an increased incidence of infections [1,28]. These findings underscore the intricate nature of critical illness-associated catabolism and emphasize the necessity for individualized feeding strategies based on patient response and nutritional risk assessment.

Guidelines and recommendations

Current Guidelines and Recommendations Regarding Nutritional Support in Critical Illness

Current guidelines and recommendations concerning nutritional support in critical illness underscore the necessity of personalized nutrition interventions to enhance patient outcomes. These guidelines prioritize early EN over PN in critically ill individuals, averting overfeeding and addressing feeding intolerance. They advocate for individualized approaches to nutrition support, considering factors such as nutrition screening, assessment, hemodynamic stability, nutrition route, tube feeding challenges, tolerance levels, and optimal calorie-protein requirements [29,30]. Furthermore, the guidelines advocate for initiating EN promptly, typically within 24-48 hours of critical illness onset, citing associated benefits and reduced complications. They emphasize the importance of a multidisciplinary team comprising nutritionists to assess and manage potential drug-nutrient interactions daily. Monitoring tolerance, assessing the adequacy of nutrition delivery, and adjusting nutritional therapy as necessary are pivotal aspects underscored in these guidelines to prevent underfeeding or overfeeding, both of which can escalate morbidity and mortality risks in critically ill patients [29,30].

Incorporating Permissive Underfeeding Into Clinical Guidelines

Incorporating permissive underfeeding into clinical guidelines for critically ill patients necessitates thoroughly considering the latest evidence regarding its efficacy and potential benefits. Recent systematic reviews and meta-analyses have indicated that permissive underfeeding, entailing 40-60% of calculated caloric requirements, holds promise in reducing ICU mortality, mitigating gastrointestinal adverse events, and shortening mechanical ventilation duration [1,26]. However, it's imperative to acknowledge that while permissive underfeeding may positively influence specific outcomes, overall mortality rates may not experience significant improvement. When integrating permissive underfeeding into clinical guidelines, it's critical to underscore the importance of personalized nutrition support tailored to individual patient needs. Guidelines should advocate for early EN over PN, primarily focusing on averting overfeeding and addressing feeding intolerance. Monitoring tolerance, evaluating the adequacy of nutrition delivery, and making necessary adjustments to nutritional therapy emerge as key components in incorporating permissive underfeeding into clinical practice [27]. Moreover, guidelines should highlight the significance of identifying patients at high nutrition risk who stand to benefit from specialized nutrition support, mainly through EN. Recommendations should emphasize the prompt initiation of nutrition support and gradual progression toward nutritional goals, all while ensuring continuous monitoring and adjustment to prevent complications from underfeeding or overfeeding. Additionally, guidelines should advocate for further well-designed, RCTs to validate conclusions concerning the impact of permissive underfeeding on patient outcomes [1,26].

## Conclusions

In conclusion, this comprehensive review has shed light on permissive underfeeding as a nuanced nutritional strategy for critically ill patients. Through exploring its physiological rationale, benefits, and limitations, it has become evident that permissive underfeeding offers a controlled approach to calorie provision that may mitigate the risks associated with overfeeding while still meeting essential metabolic needs. However, implementing permissive underfeeding requires careful consideration of patient selection criteria, close monitoring of nutritional status, and collaboration among multidisciplinary teams. The implications for clinical practice are significant, as healthcare professionals must weigh the potential benefits of permissive underfeeding against its potential drawbacks, tailoring nutritional strategies to individual patient needs. Future research should focus on refining the optimal parameters for permissive underfeeding, assessing its long-term impact on clinical outcomes, and exploring synergistic effects with other interventions. By addressing these areas of uncertainty, we can advance our understanding of permissive underfeeding and its role in optimizing patient outcomes in critical care settings.
